# Joint mouse–human phenome-wide association to test gene function and disease risk

**DOI:** 10.1038/ncomms10464

**Published:** 2016-02-02

**Authors:** Xusheng Wang, Ashutosh K. Pandey, Megan K. Mulligan, Evan G. Williams, Khyobeni Mozhui, Zhengsheng Li, Virginija Jovaisaite, L. Darryl Quarles, Zhousheng Xiao, Jinsong Huang, John A. Capra, Zugen Chen, William L. Taylor, Lisa Bastarache, Xinnan Niu, Katherine S. Pollard, Daniel C. Ciobanu, Alexander O. Reznik, Artem V. Tishkov, Igor B. Zhulin, Junmin Peng, Stanley F. Nelson, Joshua C. Denny, Johan Auwerx, Lu Lu, Robert W. Williams

**Affiliations:** 1 Department of Genetics, Genomics and Informatics, University of Tennessee Health Science Center, Memphis, Tennessee 38163, USA; 2 St Jude Proteomics Facility, St Jude Children's Research Hospital, Memphis, Tennessee 38105, USA; 3 Laboratory of Integrative and Systems Physiology, School of Life Sciences, École Polytechnique Fédérale de Lausanne, Lausanne 1015, Switzerland; 4 Department of Medicine, University of Tennessee Health Science Center, Memphis, Tennessee 38163, USA; 5 Department of Biomedical Informatics, Vanderbilt University School of Medicine, Nashville, Tennessee 37232, USA; 6 Department of Human Genetics, University of California, Los Angeles, California 90095, USA; 7 Molecular Resource Center, University of Tennessee Health Science Center, Memphis, Tennessee 38163, USA; 8 Gladstone Institutes, San Francisco, California 94158, USA; 9 Division of Biostatistics and Institute for Human Genetics, University of California, San Francisco, California 94158, USA; 10 Animal Science Department, University of Nebraska, Lincoln, Nebraska 68583, USA; 11 Joint Institute for Computational Sciences, University of Tennessee—Oak Ridge National Laboratory, Oak Ridge, Tennessee 37831, USA; 12 Department of Medicine, Vanderbilt University School of Medicine, Nashville, Tennessee 37232, USA

## Abstract

Phenome-wide association is a novel reverse genetic strategy to analyze genome-to-phenome relations in human clinical cohorts. Here we test this approach using a large murine population segregating for ∼5 million sequence variants, and we compare our results to those extracted from a matched analysis of gene variants in a large human cohort. For the mouse cohort, we amassed a deep and broad open-access phenome consisting of ∼4,500 metabolic, physiological, pharmacological and behavioural traits, and more than 90 independent expression quantitative trait locus (QTL), transcriptome, proteome, metagenome and metabolome data sets—by far the largest coherent phenome for any experimental cohort (www.genenetwork.org). We tested downstream effects of subsets of variants and discovered several novel associations, including a missense mutation in fumarate hydratase that controls variation in the mitochondrial unfolded protein response in both mouse and *Caenorhabditis elegans*, and missense mutations in *Col6a5* that underlies variation in bone mineral density in both mouse and human.

## Abstract (web summary)

Phenome-wide association is a novel method that links sequence variants to a spectrum of phenotypes and diseases. Here the authors generate detailed mouse genetic and phenome data which links their phenome-wide association study (PheWAS) of mouse to corresponding PheWAS in human.

## Graphical abstract

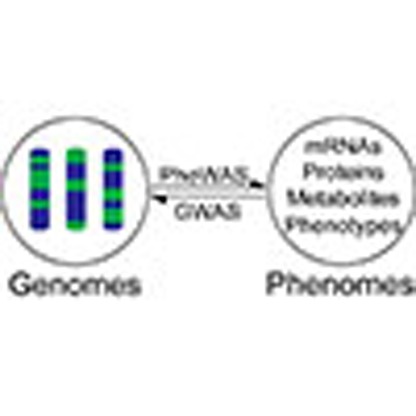

Identifying sequence variants that control or modify sets of linked phenotypes is fundamental to understanding the molecular basis of both Mendelian and complex traits^1^,^2^,^3^,^4^. A variety of reverse genetic approaches to induce loss- and gain-of-function have been used to causally tie DNA variants to discrete phenotypes^5^. However, reverse genetics presents two challenges. The first is evaluating a potentially broad spectrum of phenotypes, biomarkers and endophenotypes that are downstream of sequence variants at different stages of development, in different tissues and cells, and under different conditions. The second is evaluating the impact of these variants across different genetic backgrounds that influence trait penetrance. Phenome-wide association studies (PheWAS) address both challenges^6^,^7^.

To establish the first murine resource for phenome-wide association, we have used a large cohort of recombinant inbred strains—the BXD family—that were generated by crossing two fully inbred parental strains—C57BL/6J (B6) and DBA/2J (D2). This family consists of ∼150 isogenic lines, and as we show here, this family segregates for over five million common variants and ∼12,000 missense mutations. To accompany these genomic data, we assembled a high-coverage phenome with over 4,500 quantitative metabolic, physiological, pharmacological, toxicological, morphometric and behavioural phenotypes, along with linked references to the primary literature. This BXD phenome also includes ∼90 large open-access expression quantitative trait locus (eQTL) studies generated over the past decade as well as recent metagenomic, metabolomics and proteomic components^8^,^9^,^10^. Roughly half of the eQTL data sets are experimental, developmental or related to aging. Almost all phenome data types and genome sequence are accessible online as a companion to this paper.

The impact of ∼12,000 potentially damaging sequence variants was evaluated by systematically scanning them against the phenome at molecular, cellular and behavioural levels. We discovered new associations, some of which were subsequently validated in a large human clinical cohort^6^,^7^. For example, missense mutations in *Col6a5* were linked to variation in bone mineral density in both mouse and human. However, downstream effects of allelic variants with presumed deleterious effects on gene expression or protein structure are often small or undetectable. This may often be due to a lack of technical sensitivity and power, or due to molecular and developmental compensation.

## Results

### Phenomes

**Figure 1:**
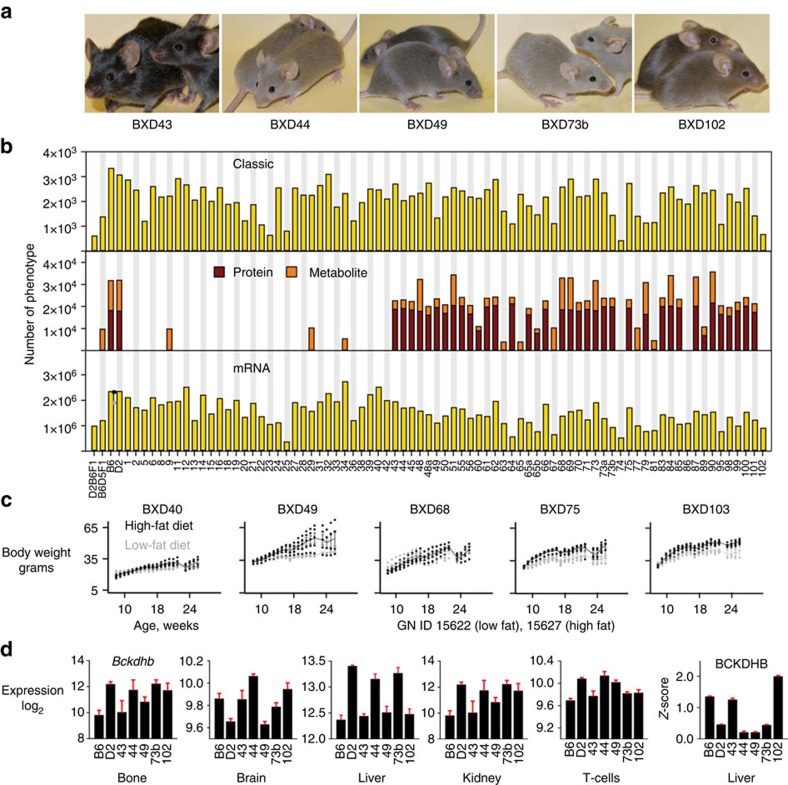
Overview of phenome data for the BXD cohort. (**a**) Five pairs of isogenic BXD cohort strains—BXD43 to BXD102. There are now approximately 100 readily available BXD strains and another 50 that are almost fully inbred. Almost all current phenome data is restricted to the parents, F1 hybrids (B6D2F1 and D2B6F1) and BXD1 through BXD102. (**b**) Phenome data categorized by type, including classic phenotypes (top and see comprehensive definitions in Supplementary Data 1), metabolic and proteomic trait data (middle), and independent mRNA expression assays (bottom, *n*=86 unique eQTL data sets, see Supplementary Note 1 Summary Expression Phenome). (**c**) Body weight data for BXD strains on high-fat (grey) and low-fat (black) diets taken from Wu *et al*.^10^. (**d**) Expression of *Bckdhb* mRNA and its protein in six tissues for the five BXD strains. Protein data from Wu *et al*.^10^

Phenome data were generated using a large cohort of recombinant inbred strains—the BXD family—that was derived by crossing two fully inbred parents—C57BL/6J (B6) and DBA/2J (D2). Members of the BXD family collectively segregate for all sequence variants that distinguish the two parents—and in this cross these are by definition common variants. There are also interesting rare but still undefined alleles unique to each family member. The level of both genetic and phenotypic variation between parents and among the strains is high (Fig. 1a). This BXD phenome includes ∼4,500 quantitative metabolic, physiological, pharmacological, toxicological, morphometric and behavioural phenotypes (Fig. 1b). These traits are almost all quantitative and have been systematically grouped into 15 major phenotype categories (Supplementary Data 1). We have also generated and assembled a large molecular phenome that includes expression phenotypes from ∼90 large open-access eQTL studies generated over the past decade (Fig. 1b and Supplementary Data 2). On average 1.5 × 10^6^ mRNA, 1.7 × 10^4^ proteomic and 6.8 × 10^3^ metabolomic assays are available per strain (Fig. 1b). Most phenotypes vary markedly across strains within the family. For example, effects of high-fat and low-fat diets on adult body weight vary substantially across genotypes (Fig. 1c). Similarly mRNA and protein expression of, for example, *Bckdhb* and many other mRNA, proteins, and metabolites vary greatly (Fig. 1d)^10^. The online availability of well-organized classic and molecular traits from the BXD family (see www.genenetwork.org) provides the foundation for multiscalar phenome scans of any putatively functional sequence variant.

The human phenome used in this study is a large electronic health record (EHR-linked cohort, BioVU https://victr.vanderbilt.edu/pub/biovu/). BioVU currently contains >190,000 DNA samples linked to de-identified medical records to provide a large, clinically relevant human resource to study genotype–phenotype associations; 29,722 of these individuals have extant exome variant data, which was used for matched mouse-to-human PheWAS in this study.

### Whole-genome sequencing and variant detection

**Figure 2:**
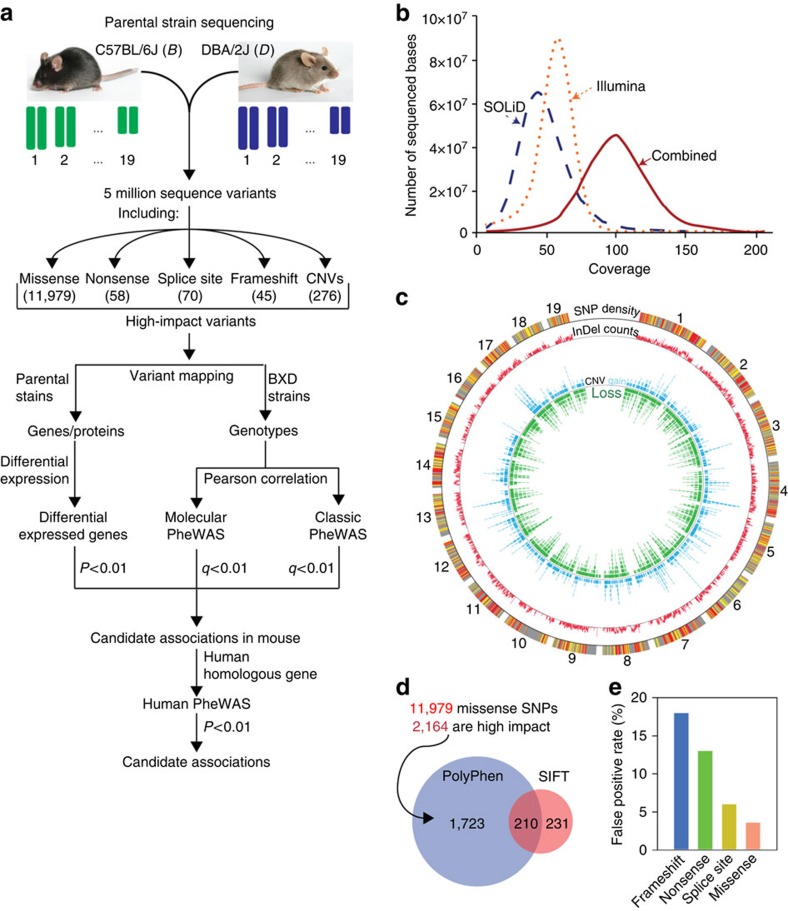
Overview of experimental phenome-wide association. (**a**) Workflow for the PheWAS analysis. D2 genome was sequenced using Illumina and SOLiD platforms and aligned to the reference genome. Approximately five million sequence and structural variants were identified. A set of high-impact variants including 11,979 missense, 58 nonsense, 70 splice site, 45 frame shift and 276 CNVs spanning at least one gene was used for classic and molecular PheWAS analyses. (**b**) Whole-genome read coverage. The distribution was generated using combined mapped reads from Illumina and SOLiD, and mapped reads from Illumina and SOLiD platforms individually. (**c**) Circos plot of sequence and structure variants segregating in the BXD cohort. The outmost circle represents SNP density per 100 kb window (black at the lowest density and orange at the highest density). The second circle represents indel density per 100 kb. The innermost circle represents CNVs. Blue and green ticks indicate D2 losses and gains, respectively. (**d**) Venn diagram of missense variants predicted to be deleterious by SIFT and PolyPhen2. (**e**) False positive rate of strong variants. A small subset of each type of variants was selected for validation by Sanger resequencing.

To obtain accurate information on sequence variants across the BXD family (Fig. 2a), we generated ∼8.26 billion reads using six paired-end libraries with different insert sizes from the D2 parent using two sequencing platforms. A total of 4.5 billion reads (262 Gb nucleotides) were aligned to the genome of the other parent of the BXD family (B6) that serves as the mouse reference genome. The mouse genome consists of ∼2.6 Gb, and we generated ∼100-fold coverage (Fig. 2b and Supplementary Data 3) and sequenced 99.96% of the reference genome excluding gaps and regions of low complexity.

The parents of the BXD cohort differ at 4.8 million single-nucleotide polymorphisms (SNPs) (Fig. 2c) at a high-confidence threshold, including 4,160,570 extracted from the SOLiD platform and 4,090,000 SNPs from Illumina (Supplementary Fig. 1a). Recently, Keane *et al*.^4^ generated ∼25-fold coverage of the D2 genome using the Illumina platform. We found a reasonably high concordance (94.4%) in SNP detection between Illumina data and that of Keane *et al*. (Supplementary Fig. 1b). The distribution of SNPs across functionally distinct genomic regions is provided in Supplementary Fig. 1c and Supplementary Note 1. We resequenced a subset of 262 platform-specific SNPs. False positive rates (FPR) were 2.34% and 3.73% for SOLiD and Illumina platforms, respectively. Assuming that all 3,375,198 SNPs detected by both systems are valid, the FPR are 0.44% for SOLiD and 0.65% for Illumina.

We defined 35,068 coding SNPs (cSNPs), of which 23,089 are silent (synonymous) and 11,979 are missense (non-synonymous) (Supplementary Data 4). Approximately 16% and 4% of non-synonymous SNPs have potentially deleterious effects on protein function as assessed using Polymorphism Phenotyping 2 (PolyPhen 2) (ref. 11) and Sorting Intolerant From Tolerant (SIFT) (ref. 12), respectively (Supplementary Data 4). Approximately 2% (210 SNPs) were defined as deleterious by both algorithms (Fig. 2d and Supplementary Data 4). We identified 58 nonsense SNPs in 53 genes, including 42 stop codon gains and 16 stop codon losses in D2 (Supplementary Data 5). The functional consequence of 210 deleterious missense variants and 58 nonsense variants were further evaluated by comparative genomic analysis (Supplementary Data 6 and 7 and Supplementary Note 1). In addition, 79 missense variants were confirmed by mass spectrometry-based proteomics (Supplementary Data 8 and Supplementary Note 1).

Most SNPs (98%) occur within noncoding regions (noncoding SNPs, ncSNPs). An impact score of each ncSNP was calculated and used for prioritization (Supplementary Fig. 1d, Supplementary Data 9 and Supplementary Note 1). Variants at splice sites result in production of non-functional or abnormal proteins and are known to contribute to diseases^13^. We detected 70 SNPs that changed conserved bases at splice sites, including 29 acceptor sites (GT) and 41 donor sites (AG) (Supplementary Data 5). In addition, 26 of the ncSNPs are predicted to alter processed miRNA sequence (Supplementary Data 10 and Supplementary Note 1).

Insertions and deletions (indels) in coding sequences can be highly disruptive, especially when they introduce frameshift mutations. We found that most small indels (98.74%) are in introns or intergenic regions, but 542 small deletions and 641 small insertions are in coding exons (Supplementary Fig. 2 and Supplementary Note 1). The small coding indels are enriched in trinucleotides, which account for 32% of small coding deletions and 38% of small coding insertions. Of the remaining coding indels, 45 are predicted to result in frameshift mutations through deletions (25) or insertions (20) (Supplementary Data 5).

Sequences from SOLiD and Illumina platforms were combined to accurately detect copy number variants (CNVs). We detected 16,817 CNVs, consisting of 4,296 gains and 12,521 losses (Fig. 2c) with an average length of 34.9 and 56.7 kb, respectively (Supplementary Fig. 3). Of copy number gains relative to the B6 genome, 79 cover 101 genes completely, while 300 cover one or more coding exons in 279 genes. Of the losses, 197 cover 259 genes completely, while 993 cover one or more coding exons in 276 genes.

All the sequence variants detected are summarized in Supplementary Data 11. FPR of each type of variants is shown in Fig. 2e, and detailed information is provided in Supplementary Note 1. The functionally important variants (that is, nonsense, missense, splice site, frameshift and CNVs) were selected for subsequent PheWAS analysis.

### Phenome-wide association analysis in mouse

We used 3,805 genotypes that represent distinct haplotype blocks in the BXD family to perform PheWAS against 4,230 classic traits as well as 602,746 endophenotypic traits from 16 distinct tissues (Fig. 1a and Supplementary Data 1 and 2). This analysis yielded ∼14 million genotype-to-phenome correlations and ∼2.0 billion genotype-to-endophenotype correlations. A total of 95 genotypes are significantly associated with 321 phenotypes, corresponding to 108 phenotypic groups, at a stringent *q* value threshold of <0.01 (Supplementary Data 12). In addition, we performed differential expression analyses between the B6 and D2 strains for each association by using transcripts from 16 tissues (Supplementary Data 2) and proteins from hippocampus (Supplementary Data 13).

We interrogated the associations for 12,420 functionally important variants, including 11,979 missense, 58 nonsense, 70 splice site and 45 frameshift mutations, and 276 CNVs, by mapping these variants to the nearest genotype markers within ±1 Mb. We found that 650 functionally important variants were associated with 97 classic phenotypes, including 634 missense variants that were associated with 62 phenotypes (Supplementary Data 14).

### Examples of variant-phenome association

Among 321 classic phenotypic associations meeting a stringent *q* value threshold of<0.01 (Supplementary Data 12), a few variants, such as those in *Gpnmb*, *Comt* and *H2-B1*, have been associated previously with disease^14^,^15^,^16^,^17^ using traditional forward genetics approaches (Supplementary Data 15), but the vast majority of variants have not been previously linked to any phenotype. Here, we provided four PheWAS examples, including two missense variants (*Fh1* and *Col6a5*), a nonsense variant *(Ahr)* and a CNV (a region covering both *Alad* and *Hdh3)*. In addition, we also provided three other examples in the Supplementary Note 1, including a missense variant in *Entpd2* (Supplementary Fig. 4), a noncoding variant in *Hcfc1r1* (Supplementary Fig. 5), and frameshift variant in *Pcm1* (Supplementary Fig. 6).

**Figure 3:**
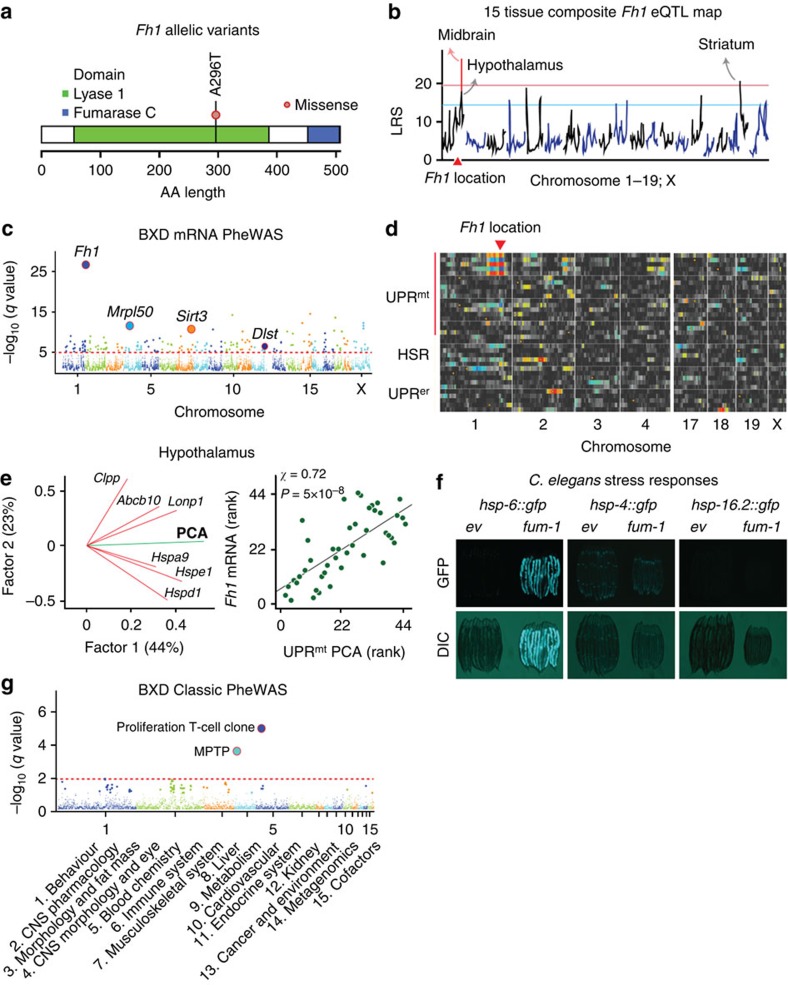
Association analysis for a missense variant in *Fh1*. (**a**) Structure of the *Fh1* gene showing lyase 1 and fumarase c domains, the former of which contains a missense mutation. (**b**) Combined eQTL mapping of *Fh1* mRNA across 15 tissues. The eigenvalues associated with the first principal component map to *Fh1* with a likelihood ratio statistic of >20. The solid red line represents genome-wide significance. The red triangle indicates the genomic position of *Fh1*. (**c**) Manhattan plot of an expression phenome scan of molecular traits linked to the *Fh1* locus in midbrain. The *y*-axis shows the −log_10_ *q* values of association, and the *x*-axis shows positions of 55,681 assays generated using Agilent SurePrint array. (**d**) QTL heat map of mRNAs involved in the unfolded protein response. The *x*-axis lists mouse chromosome numbers. Each horizontal line represents the QTL map for a single transcript in midbrain. Transcripts are grouped into three major categories—genes involved in the canonical UPR^mt^, cytoplasmic heat shock response (HSR) and unfolded protein response in the endoplasmic reticulum (UPR^er^). A subset of UPR^mt^ genes at the top are strongly modulated by the *Fh1* locus on Chr 1 (the intense colours to the upper left). In contrast, none of the UPR^er^ subsets are modulated by *Fh1*. (**e**) Principal component analysis plot for six UPR^mt^ transcripts (left). The first two components explain ∼67% of the variance in expression in hypothalamus. There is significant correlation between UPR^mt^ expression and *Fh1* (*P*=5 × 10^−8^; Pearson product-moment correlation coefficient) (right). (**f**) Validation that fumarate hydratase selectively controls the UPR^mt^ in *C. elegans.* The left-most pair of images demonstrates effects of the *fum-1* RNAi knockdown on *hsp-6::gfp* signal—a marker of UPR^mt^ induction. The middle and right panels demonstrate that the *fum-1* knockdown does not induce either the UPR^er^ (*hsp-4::gfp)*, or the cytoplasmic heat shock response (*hsp-16.2::gfp*). (**g**) Manhattan plot of a phenome scan. The *y*-axis shows the −log_10_ *q* values of ∼4,230 phenotypes, and the *x*-axis shows the 15 phenotypic categories.

The first example is a missense variant (A296T; *rs32536342*) in the fumarate hydratase mitochondrial enzyme located on chromosome 1 at 175.60 Mb (*Fh1*; Fig. 3a). *Fh1* catalyses the hydration of fumarate to malate in the tricarboxylic acid (TCA) cycle and has been linked to renal cell cancer^18^. The missense variant in the lyase 1 domain is associated with a ∼1.4-fold effect on expression of *Fh1* across many tissues, including midbrain, hypothalamus, striatum and spleen (Fig. 3b). This variant is strongly associated with *Fh1* mRNA expression, as well as the expression of other mitochondrial genes, including *Mrpl50*, *Sirt3* and *Dlst* (Fig. 3c). Expression PheWAS shows that the *Fh1* locus modulates mRNA expression levels of 113 mitochondrial proteins, in addition to eight genes linked to renal necrosis, and seven genes involved in mTOR signalling, consistent with the known role of *FH1* in renal cancer (Supplementary Data 16). Interestingly, four mitochondrial genes, *Hspd1*, *Hspa9*, *Clpx* and *Lonp1* that all encode components of the mitochondrial unfolded protein response (UPR^mt^) (ref. 19)—a still poorly characterized mitochondrial stress response pathway in mammals—show strong association with *Fh1* (Fig. 3d and Supplementary Data 16). There is, furthermore, a significant correlation between *Fh1* transcript levels and principal component scores of a group of UPR^mt^ genes in mouse (Fig. 3e). In contrast, no genes involved in the cytoplasmic heat shock response (HSR) or the ER unfolded protein response (UPR^er^) are associated with *Fh1*, indicating a selective association between *Fh1* and UPR^mt^ in mammals (Fig. 3d). To validate this association, we examined the phenotypic impact of the highly conserved *Caenorhabditis elegans Fh1* ortholog, *fum-1* (86% sequence similarity) on unfolded protein responses. RNAi against *fum-1* causes robust activation of the mitochondrial chaperone hsp-6 induces green fluorescent protein (hsp-6::gfp) reporter, indicative of the activation of the UPR^mt^ (Fig. 3f). The response was organelle-specific, and *fum-1* RNAi does not induce either *hsp-4::gfp* or *hsp-16.2::gfp*, reporters related to the UPR^er^ or HSR, respectively (Fig. 3f). Thus, in the BXD family, a decrease of fumarate hydratase leads to a specific mitochondrial phenotype, characterized by an UPR^mt^.

*Fh1* is also associated with two candidate phenotypes: (1) T-cell proliferation (GN ID 10237; *q*=2.6 × 10^−5^), linked previously to mitochondrial function^20^; and (2) dopamine metabolism after treatment with the mitochondrial toxin 1-methyl-4-phenyl-1,2,3,6-tetrahydropyridine (MPTP, GN ID 15151; *q*=0.005). Both traits are linked to *Fh1* along with the control of mitochondrial components of a UPR^mt^ pathway (Fig. 3g). No extant human genotype data are yet available for *FH*—the homolog of *Fh1*.

**Figure 4:**
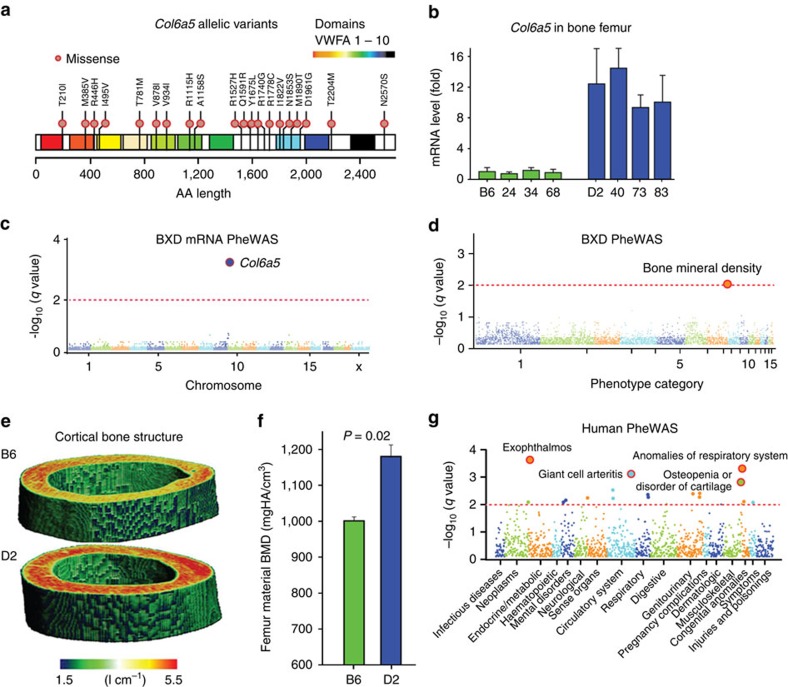
Association analysis for missense variants in *Col6a5*. (**a**) Twenty missense variants in *Col6a5* distributed across 10 von Willebrand factor A-type (vWFA) domains. (**b**) Differential mRNA expression of Col6a5 in tibias (*n*=4) measured by rtPCR. The D haplotype (blue, right) has far higher expression than the B haplotype (green) relative to *Gapdh*. (**c**) Phenome scan of *Col6a5* (rs13480398) across mRNA assays for femur. (**d**) Phenome scan of *Col6a5* (rs13480398) across classic phenotypes. (**e**) Marked difference in bone density between B6 and D2 parents. Femurs from 12-week-old mice were scanned using high-resolution micro-CT (μCT40, SCANCO Medical, Bassersdorf, Switzerland). More highly mineralized areas are indicated in red. (**f**) Difference in material bone density (*P*=0.02; two-tailed Student's *t*-test, *n*=3). (**g**) Human phenome scan for association of *Col6a5* (*rs113396273*) across BioVU.

The second example consists of a set of tightly linked missense variants in collagen 6A5 on chromosome 9 at 105.76 Mb (*Col6a5,* Fig. 4a). *Col6a5* is a variant-rich gene and contains 21 missense variants, including a radical substitution (R1778C). Quantitative RT-PCR shows higher expression of the *D* allele than the *B* allele in bone marrow (Fig. 4b). As expected, expression differences are strongly associated with the *Col6a5* locus in bone expression PheWAS (*q*=3.5 × 10^−4^, Fig. 4c). Unlike *Fh1*, the high density of linked variants in *Col6a5* means that we cannot resolve effects of single SNPs, but the scan does define effects of *B* and *D* haplotypes for *Col6a5* across the phenome. We find that this polymorphic gene is associated with differences in bone mineral density (BMD; GN ID 16532; *q*=0.037) (Fig. 4d) and quantitative micro-CT analysis confirms a marked difference in cortical BMD at the femoral midshaft between B6 (1069.6±51.4 mgHA per cm^3^) and D2 parents (1170.8±39.8 mgHA per cm^3^) (Fig. 4e,f). In humans, mutations in collagen VI are associated with a variety of musculoskeletal abnormalities^21^. We performed a matched PheWAS in human using the BioVU resource and linked *rs113396273* in exon 3 of *COL6A5* (M56T) with osteopenia and other bone and cartilage disorders (*P*=1.4 × 10^−3^; logistic regression; Fig. 4g). Like *rs113396273*, the other SNPs tested in *COL6A5* demonstrated similar patterns of associations including respiratory abnormalities and giant cell arteritis.

**Figure 5:**
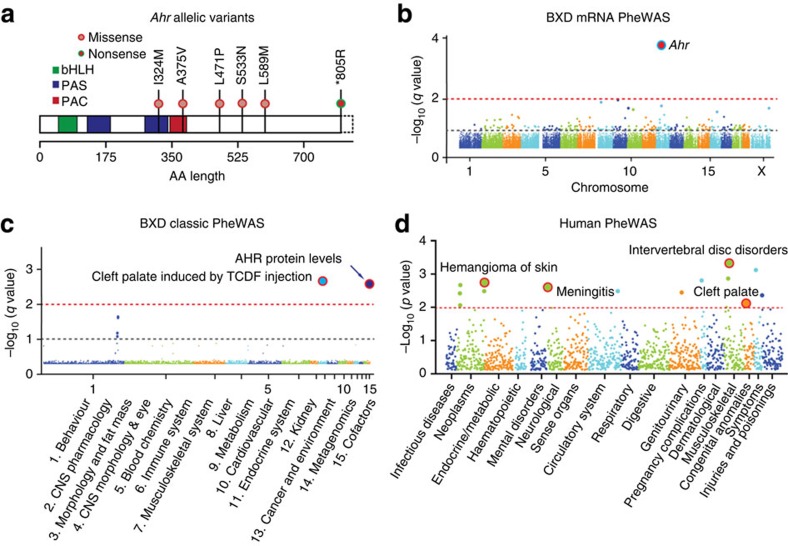
Association analysis for nonsense variant in *Ahr*. (**a**) Structure of the *Ahr* gene showing three domains with a nonsense mutation and five missense mutations. The nonsense mutation (*805R) leads to loss of the stop codon, and the addition of 43 C-terminal amino acids. Dotted rectangle to the right is the extended coding region in the *D* haplotype. (**b**) Expression phenome scan of *Ahr* (*rs3711448*) across liver mRNA levels in the BXDs. (**c**) Phenome scan of *Ahr* across classic phenotypes in the BXD strains. Both AHR protein level and cleft palate induced by TCDF injection are strongly linked to *Ahr*. (**d**) Manhattan plot showing the association in human between SNP (*rs2066853*) in *AHR* and classic phenotypes. The cleft palate phenotype is also associate with *AHR* in human clinical cohorts.

The third example is a high-impact nonsense variant—a loss of stop codon in the *D* allele of *Ahr. Ahr* is an important transcription factor that modulates P450 gene expression in response to xenobiotics such as dioxin^14^. Although the effects of this SNP on protein length are already known^22^ (Fig. 5a), the pleiotropic consequences of this mutation have not been evaluated. This variant is significantly associated with mRNA (*q*=1.7 × 10^−3^; Fig. 5b) and protein abundance of *Ahr* in liver (*q*=0.0085; Fig. 5c). Classic PheWAS linked this variant to the frequency with which cleft palates is induced by 2,3,7,8-tetrachlorodibenzofuran injection (GN ID 10714; *q*=3.2 × 10^−3^) (Fig. 5c). *Ahr* variants have also been definitively linked to differences in locomotor activity^17^. Consistent with the results of the BXD PheWAS, a matched PheWAS in humans using BioVU links *rs2066853* in *AHR* with cleft palate (*P*=0.012; logistic regression; Fig. 5d).

**Figure 6:**
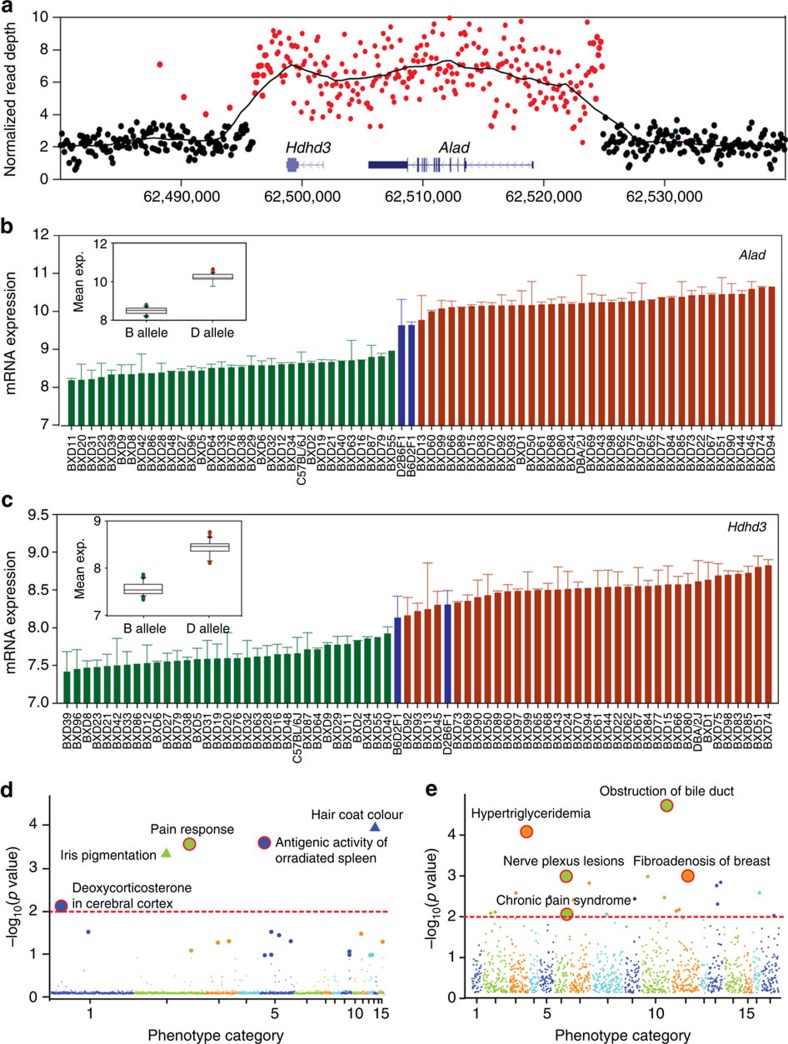
Association analysis for CNV covering *Alad* and *Hdhd3*. (**a**) The CNV region for *Alad* and *Hdhd3* derived using read-depth information from genome sequencing. Red dots represent at least a two-fold increase in coverage compared with the reference genome. The *x*-axis shows the reference genomic position of the CNV. Two gene models (that is, *Hdhd3* and *Alad*) are shown in the CNV plot. (**b**,**c**) Rank ordered mean expression levels of *Hdhd3* and *Alad* across 67 BXD strains, their parental strains, and F1 crosses. Expression values are normalized on a log2 scale (mean±s.e.m.). Strains with *D* alleles (red) have higher levels of *Alad* and *Hdhd3* compared with *B* alleles (green). F1 hybrids (blue) are intermediate. The comparison between *B* and *D* alleles for *Alad* and *Hdhd3* are shown in an inset boxplot. (**d**) The phenome scan of the BXD cohort highlights several interesting potential phenotypes including pain response (thermal nociception), brain deoxycorticosterone levels, and antigenic activity in the spleen. Two triangles represent pigmentation traits that we know are associated with a variant in the linkage disequilibrium block. (**e**) Manhattan plot obtained after phenome scan of the BioVU EHR data showing the association in humans between a SNP (*rs1800435*) in *ALAD* and chronic pain syndrome and several other classic phenotypes.

In the final example, we tested the effect of CNVs on gene expression and phenotypes. A CNV region on chromosome 4: 62.49–62.52 Mb that spans both *Alad* and *Hdhd3—*is interesting and involves a 4 × expansion in strains with the *D* haplotype. The 30 kb CNV is otherwise identical by descent (Fig. 6a). This CNV is linked with high variation in mRNA expression of *Alad* and *Hdhd3* in multiple brain regions (Fig. 6b,c), lung (*q*=2.1 × 10^−7^), eye (*q*=1.3 × 10^−10^) and liver (*q*=9.2 × 10^−4^). Quantitative proteomics of hippocampus confirms significant upregulation (ALAD 2.3-fold, *P*<0.01, HDHD3 1.5-fold, *P*<0.01, see Supplementary Data 13). The CNV expansion of *Alad* and *Hdhd3* is strongly linked to two classic phenotypes: pain response (GN ID 11307; *q*=7.8 × 10^−3^) and deoxycorticosterone levels in cerebral cortex (GN ID 12568; *q*=2.6 × 10^−4^) (Fig. 6d). A matched phenome scan in human demonstrates that *rs1800435* in *ALAD* is associated with chronic pain (*P*=2.2 × 10^−2^; logistic regression) (Fig. 6e).

### Phenotypic resilience

**Figure 7:**
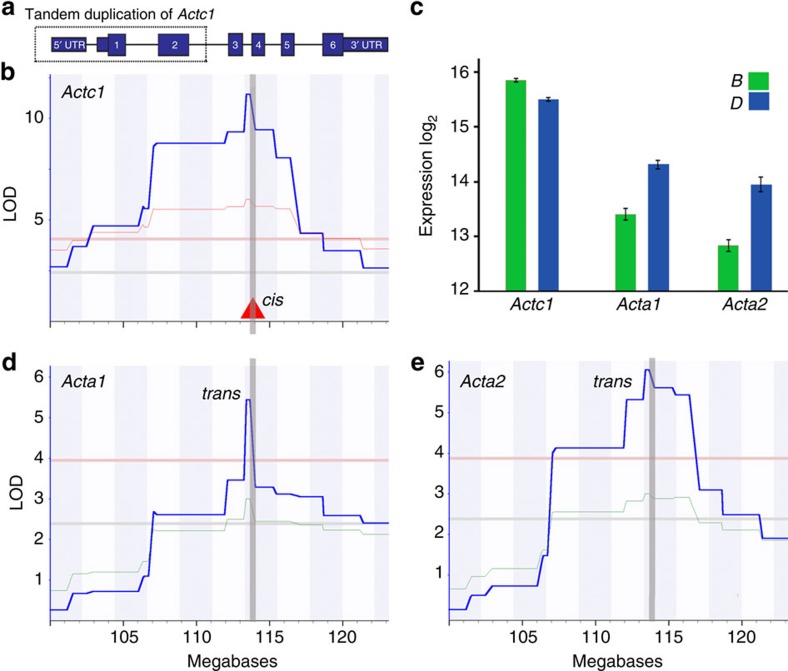
*Compensation for* Actc1 *loss by upregulation of* Acta1 *and* Acta2. (**a**) Structure of the cardiac actin gene, *Actc1*, and the tandem duplication (dotted box) in strains with the *D* haplotype. (**b**) *Actc1* expression in heart is controlled by a strong *cis*-eQTL that corresponds precisely to the location of the *Actc1* gene on Chr 2 at 114 Mb (solid red triangle). *X*-axis represents the megabase coordinate on Chr 2 while the *y*-axis represents the LOD linkage score. Thin horizontal lines provide genome-wide significance thresholds (upper red line at *P*<0.05 and lower grey line at *P*<0.63). (**c**) Mean expression of *Actc1, Acta1* and *Acta2* highlights the partial compensation for *B* haplotypes (green, *n*=24) and *D* haplotypes (blue, *n*=12). Expression values are normalized on a log2 scale (mean±s.e.m.). Heart expression data is from GeneNetwork data set GN485 (*EPFL/LISP BXD CD-HFD Heart Affy Mouse Gene 2.0 ST (Jan14) RMA*). (**d**,**e**) Both skeletal muscle and smooth muscle actins (*Acta1* and *Acta2*) also map precisely to *Actc1*.

One surprising finding is that a large proportion of genes with variants that we initially believed would have high phenotypic impact failed to associate with any classic phenotypes, or even with molecular endophenotypes. Among 41 confirmed nonsense variants with high predicted impacts, 18 nonsense variants failed to associate with any endophenotypes (across scans of 16 transcriptome data sets in different tissues) or with classic phenotypes at *q*<0.01. However, complete inactivation of four of these genes—*Scn5a, Aimp1*, *Peli3* and *Dlgap5*—is known to cause severe phenotypes (MGI database, www.informatics.jax.org). Inactivation of *Scn5a* reduces embryo size and is associated with abnormal cardiovascular system function^23^. Complete inactivation of *Aimp1* produces delayed wound healing and decreased inflammatory response^24^. The other two genes with nonsense mutations, *Peli3* and *Dlgap5*, are linked to decreased viral infection^25^ and female infertility^26^, respectively. Failure to detect associated phenotypes could be interpreted as false negative results or inadequate phenome coverage, but we suspect that most commonly this reflects molecular resilience that buffers the phenotype from apparently strong homozygous mutations. For example, a tandem duplication in the cardiac actin gene (*Actc1*) reduces expression of mRNA by ∼50% in hearts of strains that inherit the *D* allele (Fig. 7a–c). This duplication upregulates expression of both skeletal muscle actin (*Acta1)* and smooth muscle actin (*Acta2*) by 30 and 50%, respectively (Fig. 7c). Since variation in both of these actin transcripts maps precisely to *Actc1*, it is clear that this compensation is ultimately caused by the duplication (Fig. 7d,e). The depletion of cardiac actin has already been shown to be associated with compensatory increase in skeletal and smooth muscle actins in the mouse heart^27^. Another study has shown that *Actc1* can effectively replace *Acta1* to produce adequate function in the mouse postnatal skeletal muscle^28^.

## Discussion

Recent work has demonstrated that phenome scans are a powerful way to link sequence variants to sets of phenotypes in clinical cohorts^6^,^7^. Here we have extended this approach to a murine cohort for which we have been generating cellular and molecular traits from many tissues and cell types and for which we can generate data on gene-by-environment interactions^8^,^10^,^29^. The variety and depth of phenotype data that we have assembled over the past decade for the BXD cohort make this the largest coherent multiscalar data set for any segregating population. Of course, there are an almost unlimited numbers of ways to extend this BXD phenome—from much more extensive gene-environment interaction (GXE) studies to single-cell omics, but at the current size, the phenome is certainly large enough to explore the utility of PheWAS in an experimental population. We demonstrate that phenome scans can be effective at linking sequence variants to a range of phenotypes and can be used to identify novel and unexpected genome-to-phenome relations, or to validate hypothesized associations from independent studies. Coupling mouse and human PheWAS cohorts also shows great promise, and provides an efficient method to validate and translate key genome-to-phenome relations.

The novel associations demonstrated in this study provide insight into the genetic basis of complex traits and variation in disease susceptibility. The missense variant in *Fh1* is a case in point. A variant in *Fh1* is linked to the UPR^mt^, a protective stress pathway specific to mitochondria, and we confirmed that downregulation of *fum-1*, the *C. elegans* homolog of *Fh1*, activates the UPR^mt^. Various disturbances have been shown to induce the UPR^mt^, including treatment with paraquat, a pesticide that strongly induces reactive oxygen species^30^, activation of mitochondrial biogenesis^31^, overexpression of aggregation-prone mitochondrial proteins^32^ and interference with electron transport chain protein expression and assembly^19^,^33^. Here, we show that a purely metabolic perturbation, such as induced by loss of function of the TCA cycle component, fumarate hydratase, can activate the UPR^mt^. While we have detected a single missense variant in *Fh1*, the molecular cascade that links *Fh1* to other TCA cycle genes (that is, *Dlst*, *Sdha* and *Sdhb*) and a UPR^mt^ proteostasis regulatory loop requires additional analysis.

Despite strong functional effects of variants in humans, the minor allele frequencies are often too low to attain sufficient sample size. Murine populations such as the BXDs, the Collaborative Cross and heterogeneous stock typically have linkage disequilibrium that is at least an order of magnitude larger than in humans. Consequently, the assignment of specific causality may be erroneous. For example, in the BXD family ∼20,000 protein coding genes and 12,000 coding variants are distributed across ∼4,000 haplotype blocks. Increasing the size and genetic diversity of a reference population and the number of recombination events can improve genetic resolution, but a more effective and meaningful solution, exemplified in this study, is to exploit other mouse cohorts and human cohorts for validation and cross-species translation. For example, by having multiple phenomes for a single species, along with matched databases of segregating sequence variants, it would become practical to rapidly test the replicability of genome–phenome relations. It may soon be practical to compare the BXD phenome with that of the Collaborative Cross and other large families of RI strains. Any cohort will only segregate for a subset of possible sequence variants, and variants will often not be shared across populations or species. For this reason, conservation of gene function will be a more useful currency of exchange^34^,^35^.

While PheWAS has great potential, this approach faces several hurdles to more widespread application. The first is simply the technical and logistical challenge of generating a phenome. Intense collaborative efforts are a *sine qua non* even for the most tractable model organisms such as *Drosophila*^36^. The second is yet another example of the multiple testing problem: what is the appropriate correction given the size of the phenome and its structure? We have computed false discovery rate (FDR) *q* values at a conservative threshold and have aligned our results, when possible, with the BioVU clinical cohort. However, in both species, the selection of appropriate *q* values will depend on the purpose of studies and the relative costs of types 1 and 2 errors. Effective solutions may require adjusting thresholds based on the scope and intent of studies, as well as prior information about gene-to-phenotype relations. Alignment of phenotype associations across both humans and mice, however, adds validity to both. Very large, densely genotyped or sequenced populations will be needed to more deeply interrogate the human phenome. The third problem is linkage disequilibrium. The intervals in which sequence variants are located is a critical factor in mapping its phenotype spectrum. Pleiotropy will be inflated as a function of gene density, SNP density, and haplotype block structure. Deconvolving contributions of linked polymorphisms will, in most cases, still require independent experimental validation and, when possible, PheWAS of human cohorts.

We searched for molecular and functional consequences of ~12,000 coding variants, and were surprised that only a small fraction had strong effects on mRNA and protein expression, let alone on classic phenotypes. Is this resilience real or not? One obvious factor contributing to apparent phenotypic resilience may be inadequate depth of the phenome. Phenotypes may be detected only under specific conditions. For example, effects of the mutation in *Nnt* are much more pronounced under metabolic stress^10^. Artifactual resilience may also be caused by the presence of neighboring in-frame stop codons or splice acceptor or donor sites. For example, the loss of a stop codon in *Dlgap5* only adds two amino acids due to the presence of a tandem stop codon six nucleotides downstream. Additionally, some negative results are likely to be caused by incorrect or incomplete gene models that generate spurious high impact variants.

In contrast, genuine phenotypic resilience is likely to result from functional overlap and compensation among paralogs and other members of complex molecular networks. Even after stringently filtering both sequence data and gene models, it is clear that many strong sequence variants are successfully buffered at intermediate levels^37^,^38^,^39^. For example, a splice site mutation in *Cyp2c39* (Supplementary Table 5) inactivates this P450 enzyme but has no detectable impact on higher order phenotypes—a compelling negative result. An obvious explanation is overlap with other members of the Cyp2c cluster. The strongest exemplar of paralog buffering in our study is the mutation in *Actc1*, which causes compensatory upregulation of the expression of both *Acta1* and *Acta2* (Fig. 7). In retrospect, this buffering of genetic variation is not surprising. A large fraction of knockout mutations in mice and other well-typed species are viable and many of these do not have any known functional consequences^40^,^41^.

The combination of deep phenotyping and full genome sequencing makes it possible to reverse the polarity of genome-wide analysis and to measure the impact of defined sequence variants at many levels to biological organization—from mRNAs to metagenomic profiles and behavioural variation. The combination of experimentally tractable murine resources, such as the BXD family, with human clinical cohorts such as BioVU, is an efficient and scalable way to validate and translate genes to the linked sets of phenotypes. Even negative results can be genuinely informative, essentially an inverse of missing heritability.

## Methods

### DNA sample for sequencing

DBA/2J foundation breeding stock at generation F223 was obtained from The Jackson Laboratory (www.jax.org). Genomic DNA was isolated from livers of filial generation F224 female littermates using a QIACube and DNeasy kits (Qiagen Inc., Valencia, CA).

All work with mice as conducted under a protocol approved by the UTHSC institutional animal care and use committee.

### Libraries preparation

Six libraries were constructed. Three mate-paired libraries with insert sizes of 1, 2 and 3 kb were prepared for ligation-based sequencing (ABI SOLiD 2 and 3). Three paired-end libraries with insert sizes between 175 and 215 bp were prepared for sequencing by synthesis (Illumina GAII and HiSeq 2000). Standard protocols recommended by the manufacturers were used in all cases.

### Sequencing

Three mate-paired libraries were sequenced using ABI systems according to the vendor's protocol. Three libraries were sequenced using reagent kit V4 and Illumina systems according to the manufacture's standard protocol. Read lengths for the SOLiD2 and SOLiD3 systems were 2 × 25 bp and 2 × 50 bp, respectively.

One library was sequenced using the sequencing reagent V4 on the Illumina HiSeq 2000 following the manufacture's standard cluster generation and sequencing protocol. Sequence reads with 101 bp on both ends were extracted from images files using the GA software (version 1.4).

### Sequence alignment

SOLiD reads were aligned to the B6 reference genome using two alignment tools: Corona Lite v4.0.2 (http://www.thermofisher.com/us/en/home/technical-resources/software-downloads/solid-software.html) and the MAQ code v0.7.1 (ref. 42). Corona Lite parameters were set to allow up to two and six mismatches for 25 and 50 bp sequence fragments, respectively. The longer reads from Illumina were mapped to the reference genome using MAQ, allowing up to two mismatches in the first 24 bp of each read. All alignment files were converted into BAM format. Reads that aligned to only one location with no more than two mismatches in the first 24 bp were considered uniquely aligned.

### SNP detection

ABI Bioscope software was used to align colour-space reads and to detect SNPs against the B6 reference genome. Similarly, MAQ software was used to align reads and to identify SNPs. A threshold of three or more supporting reads and a consensus quality score >30 (Illumina) and a confidence value >0.5 (SOLiD) was used to declare SNPs. Rare heterozygous calls, presumably generated by alignment errors, were discarded.

### Indel detection by mapped and unmapped reads

Four tools were used to identify indels—Corona Lite small indels pipeline, MAQ, Pindel^43^ and BreakDancer^44^. Corona Lite was used to identify insertions of up to 3 bp and deletions of up to 11 bp using SOLiD sequence. Indels were further filtered by requiring at least three supporting reads, and indels within regions having extremely high coverage (1000 ×) were excluded. MAQ was also used to identify insertions up to 19 bp and deletions of up to 86 bp for 101 bp Illumina reads. A threshold of a minimum of three supporting reads and a minimum consensus quality of 30 was used. Rare heterozygous indels were discarded. Pindel was used to identify indels from 1 to 100 kb. Access to all of these variants is available on a custom version of the UCSC Genome Brower at http://ucscbrowser.genenetwork.org/cgi-bin/hgGateway.

### Large deletions detected by mapping distance

AB large InDel Tool (v1.0), which identifies deviation in clone insert size, was used to detect large indels from SOLiD reads. Indels were supported by at least two clones (cluster of reads) and a distance deviation greater than 6 s.d. from the normal distribution of insert size. Breakdancer^45^ was used to detect large structural indels (100–100,000 bp) from Illumina reads. The distribution of insert size from high quality (>35) paired-end reads was computed, and those greater than 6 s.d. from the mean were used to define large indels. A minimum confidence score of 90 was used by BreakDancer. All clones that deviated by more than 100 kb were discarded for both methods.

### CNV detection

Before performing CNV detection, we converted mapping data into the BAM format and removed gaps in the reference genome. CNVs were detected using an event-wise testing method based on read depth^46^. This method estimates the coverage of read depth in 100 bp non-overlapping windows and then performs significance testing. In this study, CNVs were defined as having at least 10 consecutive windows (100 bp each) with a minimum size of 1 kb. Merged events were filtered stringently at a significance level of 10^−6^.

### Genes affected by sequence and structural variants

We examined the impact of SNPs, indels, inversions, and CNVs located within annotated gene models (ENSEMBL version 60). Each variant was evaluated for overlap with gene models and annotated as 5′ UTR, coding/exon, intron, 3′ UTR and intergenic regions using in-house Python scripts (available upon request). Sequence variants were further examined and classified by types of variants, including nonsense, missense, splicing variant and frameshift. When possible, variants were also annotated by gene symbol and other accessions.

### Generating impact scores for coding and noncoding SNPs

We used two different methods—SIFT and PolyPhen—to predict whether amino acid substitutions in coding SNPs are likely to affect protein function. SIFT uses sequence homology and the physical properties of amino acids to predict impact, while PolyPhen uses sequence-based and structure-based predictive features.

The impact scores for ncSNPs consist of two parts: a functional element score and a mutation effect score. The functional element score reflects how likely it is that the SNP is located in a functional region of the genome, and the mutation effect score estimates how likely a mutation at this position is to affect the function of an overlapping region. Any ncSNP that overlaps a known functional element in the UCSC Mouse Genome Browser (mm10, GRCm38 assembly38) receives a functional element score of 1. Functional elements are defined using the following gene regulation tracks: open regulatory annotation (ORegAnno) elements, the NHGRI's bi-directional promoters, and Repressor Element 1-Silencing Transcription Factor (REST) binding sites, as well as conserved elements as predicted by the phastCons program (conservation track) (ref. 47). Furthermore, ncSNPs overlapping a predicted conserved element were assigned the element's phastCons score. The mutation score is based on the evolutionary conservation of the position in alignments with other placental mammals. The phyloP package (ref. 48) was used to estimate conservation. The ncSNP impact score is the product of the functional element and mutation effect scores. If the data required to compute the two scores were missing for one, but not the other, then the missing score was assigned a non-zero value less than the scores observed for all positions with data present. This prevents evidence for the functional relevance of a SNP from one source from being completely ignored if the other is not present. Thus, all ncSNPs with non-zero impact scores have some evidence of functional relevance.

### Comparative genomic analysis

Orthologs of murine proteins from complete genomes of 77 higher eukaryotes were obtained using BLAST searches. Genomes containing paralogs of a given gene were excluded from consideration. Multiple sequence alignments of orthologs were constructed using the LINS-I algorithm in MAFFT^49^. Amino acid substitutions in a position corresponding to a given SNP were analysed. SNPs that occur in invariable positions in mammals were considered likely to be deleterious. Non-mammalian conservation was considered as enhancing prediction strength. SNPs that occur in variable positions in mammals were considered unlikely to be deleterious. Variable positions in rodents and primates were also considered unlikely to be deleterious.

### Experimental validation for SNPs

SNPs and indels were selected for validation by traditional Sanger sequencing. Primers were designed using Primer3 (http://frodo.wi.mit.ed/primer3/). PCR assays were performed using 5 ng DBA/2J genomic DNA, 10 pmol each of forward and reverse primer in 50 μl. The following cycle parameters were used: 95 °C for 4 min; 35 cycles of 95 °C for 30 s; 55 °C for 30 s and 72 °C for 1 min; and 72 °C for 5 min. PCR products were purified with 2 μl ExoSAP-IT (Invitrogen Corporation). Sanger sequencing was performed using an ABI 3730.

### Experimental validation of indels

A total of 40 indels were selected from each chromosome for validation. These included 20 small- to medium-sized insertions (<86 bp), 20 medium-sized deletions (size >50 bp), and 20 large deletions (>100 bp) detected by Illumina GA2. Primers were designed by centring the target indel to produce amplicons that were 300–400 bp in length using Primer 3. PCR assays were performed as described above. We ran PCR-amplified genomic DNA on SDS–polyacrylamide gel electrophoresis gels. Each PCR assay was performed on DNA from B6 and D2. The sizes of the resulting PCR products were compared with the predicted size of indels.

### Detection of SNPs in Affymetrix probe-binding regions

To find probe sequences affected by variants (SNPs and small indels) within probe binding regions of the Affymetrix Mouse Genome 430 2.0 array, we mapped all probes to the mouse reference genome using Blat. Probe regions were then examined for the presence of SNPs or small indels.

### Proteome-wide quantification between the B6 and D2 strains

Adult B6 and D2 strains were used for protein quantification with three biological replicates of hippocampus. The hippocampal tissues were dissected as previously described^50^ and lysed in lysis buffer (8 M urea, 0.5% sodium deoxycholate, 50 mM HEPES and pH 8.5). In short, fixed brains were bisected along the midline. Left and right hippocampal regions were dissected under a dissecting microscope by inserting fine blunt forceps into the ventricular cavity just dorsal to the hippocampus and removing overlying cortex and callosum. The surface of the hippocampus and dentate gyrus was used to guide removal of cortex along the septotemporal axis. The exposed hippocampus and dentate gyrus was pulled free of the hemisphere in a ventral-to-dorsal direction. The dorsoanterior aspect of each hippocampus was trimmed free of septum and dorsal fornix, rolled quickly in tissue paper, and immediately weighed to the nearest 0.1 mg. The dissection includes a small part of the subiculum adjacent to CA1 and occasionally a small strand of the fimbria. For each sample, 100 μg of extracted proteins were digested with LysC (1:100, w/w) for 3 h. Samples were diluted four times with 50 mM HEPES followed by trypsin digestion (1:50, w/w) overnight at room temperature. After digestion, the samples were acidified with trifluoric acid to a pH<2 and desalted and dried in a speed vacuum. Peptides were labelled with six-plex TMT reagents (Thermo Scientific) as recommended by the manufacturer. Following labelling, the TMT-labelled samples (TMT126-TMT131) were mixed equally to generate a digest mixture which was further fractionated by high pH reverse phase liquid chromatography. Ten fractions are collected and further analysed by low pH reverse phase liquid chromatography-tandem mass spectrometry (MS/MS).

All digest mixtures were analysed on Q Exactive MS (Thermo Fisher Scientific) with one MS survey scan and up to 10 data-dependent MS/MS scans. The instrument was operated at a mass resolution of 35,000, 1e6 automatic gain control target, and 60 ms maximal ion time for MS, and a mass resolution of 17500, 10^5^ automatic gain control, 250 ms maximal ion time, 2 *m/z* isolation window and 30 s dynamic exclusion time for MS/MS. The normalized collision energy was set to 28%.

Raw data from each fraction were searched using SEQUEST (v28) against a mouse SwissProt/trEMBL database (release of 09/09/2011), concatenated with a decoy database with all the protein sequences in reverse order. Searches were performed using a 15 p.p.m. mass tolerance for precursor ions and 0.02 Da window for fragment ions, allowing up to two missed trypsin cleavage sites. Six-plex TMT tags on lysine residues and peptide N termini (+229.162932 Da) and oxidation of methionine residues (+15.99492 Da) were used for dynamic modification, and carbamidomethylation of cysteine residues (+57.021 Da) was used for static modifications.

### Organization and categorization of mouse phenome

The BXD Phenotype database has been amassed by literature review, direct data entry by our team, and by collaboration with many investigators. Data are reviewed before entry in GeneNetwork by the senior author. Phenotypes are currently split into 15 broad phenotypic categories (Supplementary Data 1). Phenome curation and description was initiated by R.W.W. and Dr Elissa Chesler in 2002 by literature review and data extraction. The early work is described briefly in Chesler *et al*.^51^,^52^. Most work over the past 5 years has been performed by two of the coauthors (R.W.W. and M.K.M.). We have used a controlled vocabulary and set of rules described here (http://www.genenetwork.org/faq.html#Q-22). Descriptions include a ‘prefix' of major biological and domain categories such as ‘central nervous system', ‘cancer biology' and ‘immune system'. These domains have been used to define major categories used in figures and graphs.

### PheWAS analysis in mice

PheWAS were performed for a total of ∼12,000 variants, including 11,979 missense, 58 nonsense, 68 splice site, 39 frameshift mutations and 276 CNVs The closest marker for each variant from a set of 3,804 genetic markers—each representing a unique haplotype block—was used to represent that variant in the PheWAS. We used 16 expression data sets representing different tissues of the BXD strains to explore the genetic basis of variation at mRNA levels. Similarly, we used 4,236 classic phenotypes from GeneNetwork.org (www.genenetwork.org) to study the association between variants and phenotypes. We calculated the *P* value of the Pearson correlation between each marker (variant) and 4,236 phenotypes and ∼40,000 transcripts for the expression data. All *P* values of correlation were calculated as a two-tailed test, and the *q* values (FDR) were calculated using QVALUE (ref. 53). We used an FDR threshold of 0.01 for associations. The analyses were performed using in-house Python scripts, and the R statistical package.

### PheWAS in humans

PheWAS for human data was performed using 29,722 individuals with Illumina HumanExome array data identified as European ancestry in the EHR and by using structure^54^. To define diseases, we mapped International Classifications of Diseases, 9th edition, (ICD9) codes from the EMR into 1,645 possible PheWAS phenotypes using methods described previously^6^. PheWAS phenotypes aggregate like ICD9 codes together (for example, type 2 diabetes codes as a specific phenotype), are hierarchical (for example, ‘inflammatory bowel disease' is a parent of ‘Crohn's disease' and ‘Ulcerative colitis'), and include logic to select controls for each case definition. We considered only phenotypes with at least 20 individuals for analysis, and required each case to have at least two ICD9 codes for a PheWAS phenotype to be considered a case (those with only one code are neither a case nor a control). Each SNP-phenotype association test was run with PLINK (ref. 55) using logistic regression adjusted for age, sex and the first three principal components as calculated by EIGENSTRAT using ancestry informative markers. Analysis was performed assuming an additive genetic model. These data were aggregated and analysed using Perl scripts and the R statistical package. A total of 1501 phenotypes were considered, for a per-SNP Bonferroni correction of 0.05/1501=3.3 × 10^−5^.

We then performed PheWAS for missense SNPs for each of the target genes from the mouse PheWAS that had minor allele frequencies >1% and passed quality control filters of SNP call rate >95% and sample call rate >99% in unrelated samples. SNPs were found for *ENPTD2* (*rs34618694*)*, COL6A5* (*rs1353613*, *rs79867908*, *rs12488457*, *rs113396273*, *rs35886424*, *rs1453241*, *rs1497312*, *rs11917356*, *rs76864445*, *rs16827497*, *rs16827168*, *rs819085*, *rs9883988* and *rs61744488*), *AHR* (*rs2066853*), *ALAD* (*rs1800435*) and *HDH3* (*rs1043836*). No SNPs were available for *FH1*.

All studies were approved by local Institutional Review Boards. Patients gave consent as part of the DNA biobanks at Group Health Cooperative, Marshfield Clinic, Mayo Clinic, Northwestern University; Vanderbilt uses an opt-out model as previously described and evaluated^56^,^57^.

### *C. elegans* experiments

*C. elegans* were cultured at 20 °C on nematode growth media agar plates seeded with bacteria. Strains were provided by the *Caenorhabditis* Genetics Center (University of Minnesota). The strains used were SJ4100 (zcIs13[hsp-6::GFP]), SJ4005 (zcIs4[*hsp-4*::GFP] and dvIs70 [hsp-16.2p::GFP+rol-6(su1006)]. RNAi constructs were isolated from the RNAi feeding library (GeneService) and experiments were carried out using standard feeding methods. The identity of each RNAi clone was verified by sequencing. RNAi treatment was started at embryonic stage. GFP was monitored in day 1 adults. Worms were immobilized with 6 mM solution of tetramisole hydrochloride (Sigma) in M9 and imaged using Nikon DS-L2 fluorescent microscope.

## Additional information

**Accession codes:** The raw sequencing data has been submitted to the NCBI Sequence Read Archive under accession number SRA009489. Variant calls for both data sets are available via a mirror site of the UCSC genome browser (ucscbrowser.genenetwork.org). Additionally, 4.875 million SNPs and 0.67 million indels have been submitted to the dbSNP database (www.ncbi.nlm.nih.gov/SNP) using the submitter handle UTHSCSEQ.

**How to cite this article:** Wang, X. *et al*. Joint mouse-human phenome-wide association to test gene function and disease risk. *Nat. Commun.* 7:10464 doi: 10.1038/ncomms10464 (2016).

## Supplementary Material

Supplementary Information Supplementary Figures 1-6, Supplementary Note 1 and Supplementary References

Supplementary Data 1 Categorized phenotypes for the BXD Cohort

Supplementary Data 2 Summary of expression datasets

Supplementary Data 3 Summary of sequencing data for the DBA/2J genome

Supplementary Data 4 Nonsynonymous SNPs segregating in the BXD family

Supplementary Data 5 Nonsense, splicing site and frameshift mutations segregating in the BXD family

Supplementary Data 6 Coding SNPs in disease-associated genes predicted to be likely deleterious by comparative genomic analysis

Supplementary Data 7 Coding SNPs identified by PolyPhen and SIFT as likely deleterious that are not supported as such by comparative genomic analysis

Supplementary Data 8 Missense variants confirmed by peptide sequencing

Supplementary Data 9 All noncoding SNPs with impact score > 0.9

Supplementary Data 10 SNPs affecting pre-miRNA

Supplementary Data 11 Summary of the number of variants in each category

Supplementary Data 12 PheWA summary for classical phenotypes

Supplementary Data 13 Differentially expressed proteins between the B6 and D2 strains

Supplementary Data 14 Summary of strong variants and associations in mouse

Supplementary Data 15 Novel and known variants between the B and D genomes associated with phenotypes in BXD Cohort

Supplementary Data 16 Genes highly associated with the Fh1 locus
