# Supplementary material for: Joint mouse–human phenome-wide association to test gene function and disease risk
Source: Nat Commun. 2016 Feb 2;7:10464. doi: 10.1038/ncomms10464 (PMC4740880; doi:10.1038/ncomms10464)
Supplement: Supplementary Information — Supplementary Figures 1-6, Supplementary Note 1 and Supplementary References [file ncomms10464-s1.pdf]

Supplementary Fig. 1. SNPs identification and comparison between the B6 and D2 genomes

(a) Overlap of SNPs detected by SOLiD and Illumina platforms. (b) Venn diagram showing comparison of the number of SNPs between our data (UTHSC) and Sanger. (c) The proportions of SNPs identified as intronic, coding sequencing region (CDS), 3' UTR, and 5' UTR. Of 4.85 million SNPs, a total of 1.55 million SNPs lying within 27,524 UCSC reference genes regions were used. (d) The impact scores of ncSNPs across different parts of genes.

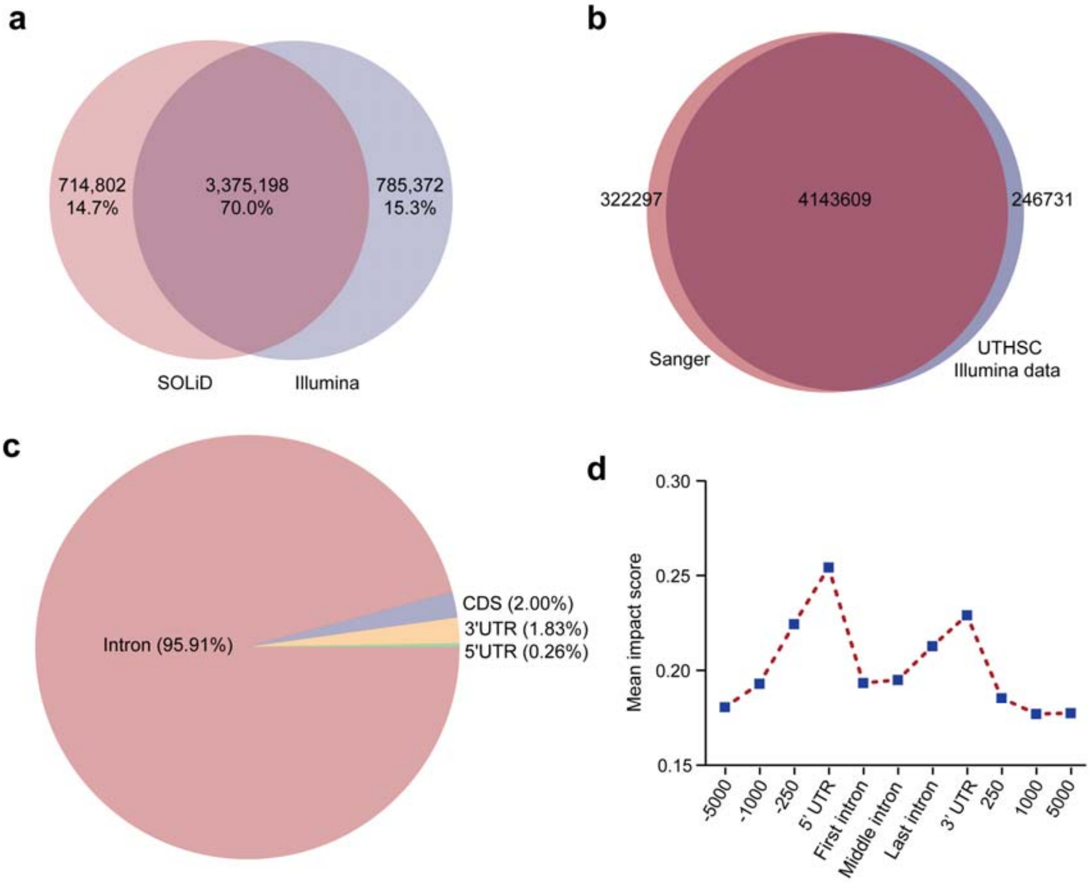

Supplementary Fig. 2. Indels identification and comparison between the B6 and D2 genomes

Distribution of (a) Deletion and (b) Insertion lengths. Two peaks in the large indels (right most top panel) represent polymorphisms in B1/B2 SINE repeats (230 bp; $\log_{10}(\text{size}) = 2.3$) and L1 LINE repeats (~7000 bp; $\log_{10}(\text{size}) = 3.9$), respectively. (c-d) Comparison of Indels detected by three algorithms: Pindel, MAQ, and SOLiD small indel pipeline. (e) Venn diagram showing comparison of the number of indels between our data (UTHSC) and Sanger.

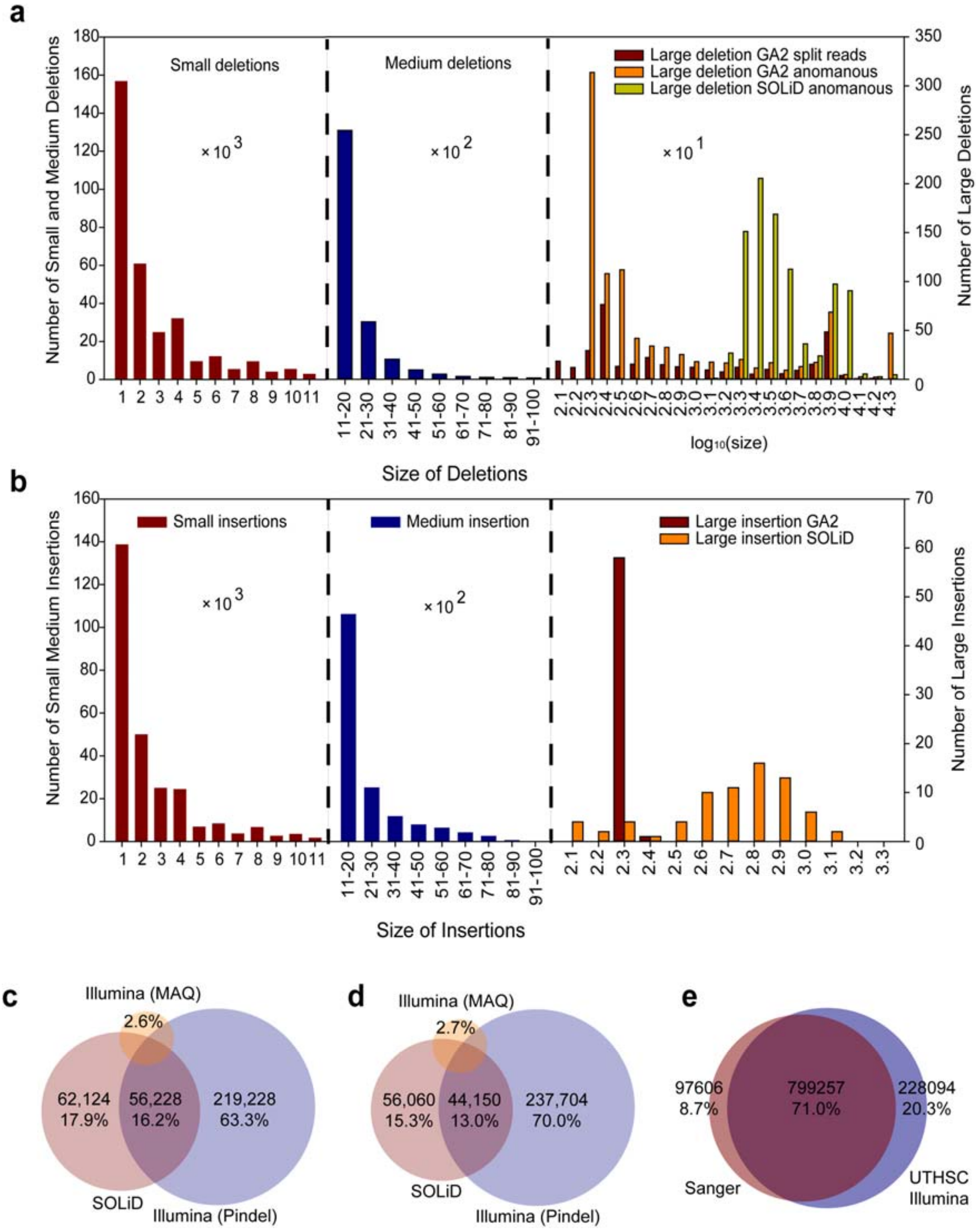

Supplementary Fig. 3. Distribution of copy number gains and losses by CNV size
The *x* and *y* axes represent size and number of CNV segments, respectively.

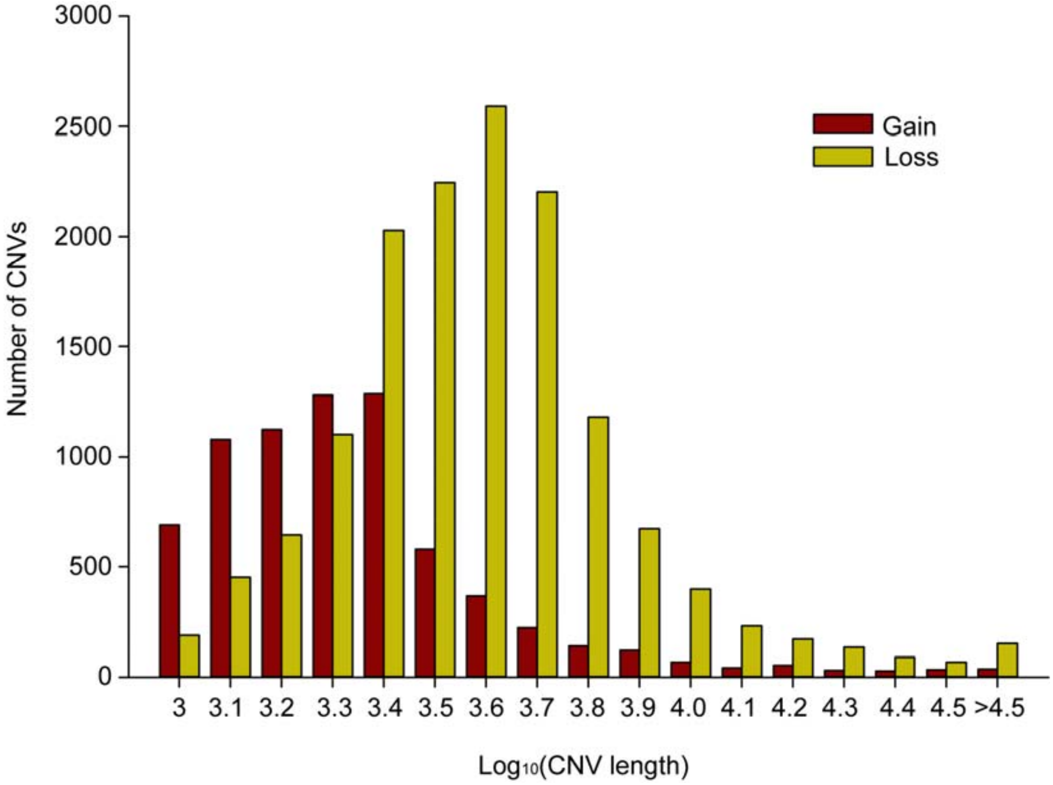

Supplementary Fig. 4. Association analysis for missense variants in *Enptd2*
(**a**) Structure of the *Entpd2* gene showing two missense mutations and GDA/CD39 family domain. (**b**) *Entpd2* expression differs at both the mRNA and protein levels between B6 and D2 strains (n = 3 replicates/genotype). (**c**) Phenome scan of *Entpd2* as in Fig 3. (**d**) Human phenome scan of *ENTPD2* across thousands of clinical records using BioVU Denny *et al.* (2013). The SNP *rs34618694* was selected for this scan. Three clinical traits related to eye diseases are highlighted. The red dotted line is at $p < 0.01$.

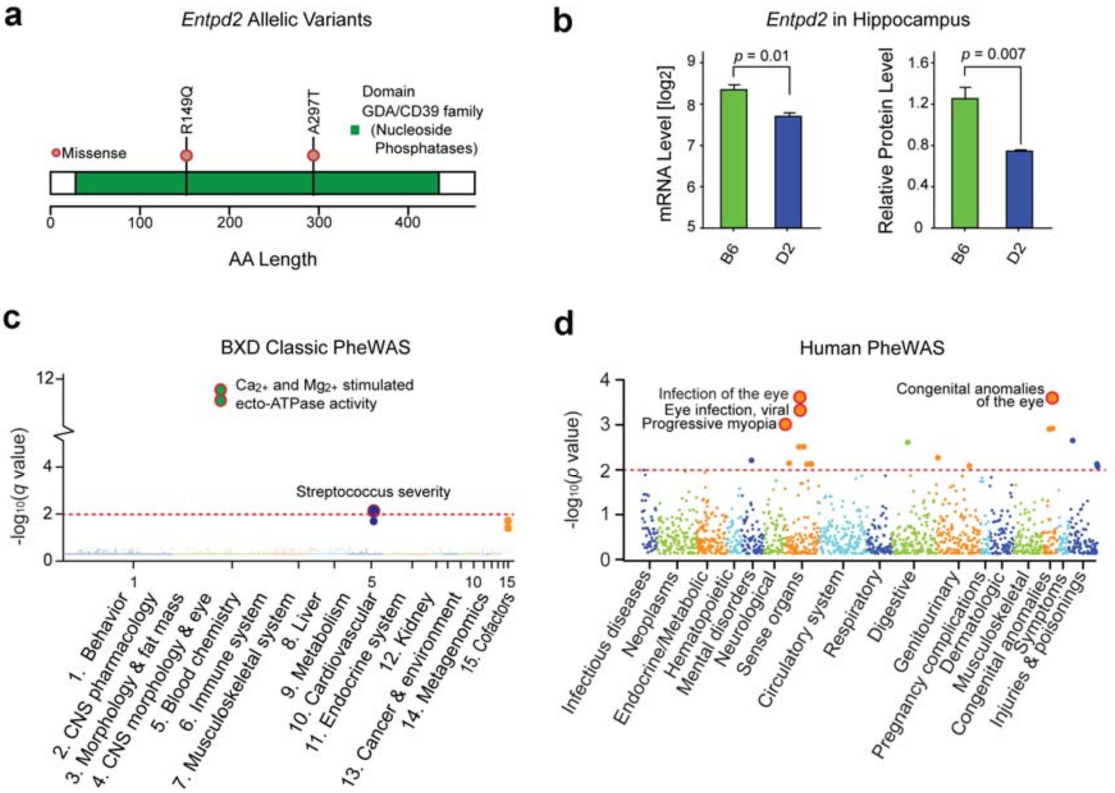

Supplementary Fig. 5. Splice site variant in *Hcfc1r1*

(a) A SNP (A/G; chr17 at 23,674,533 bp) at splice acceptor site is indicated by a vertical arrow. The reference gene structure is shown in dark red, whereas the mutated isoform with Ensembl annotation (Ensembl gene ID: ENSMUSG00000023904) is shown in dark blue. Two dotted lines represent start positions of exon 2 in reference gene structure and mutated isoforms, respectively. (b) DNA sequences in exon2 of the reference gene but not in mutated isoform are shown in peach pink. Partial common sequences are shown in dark red. (c) Protein sequence conserved among other mammals.

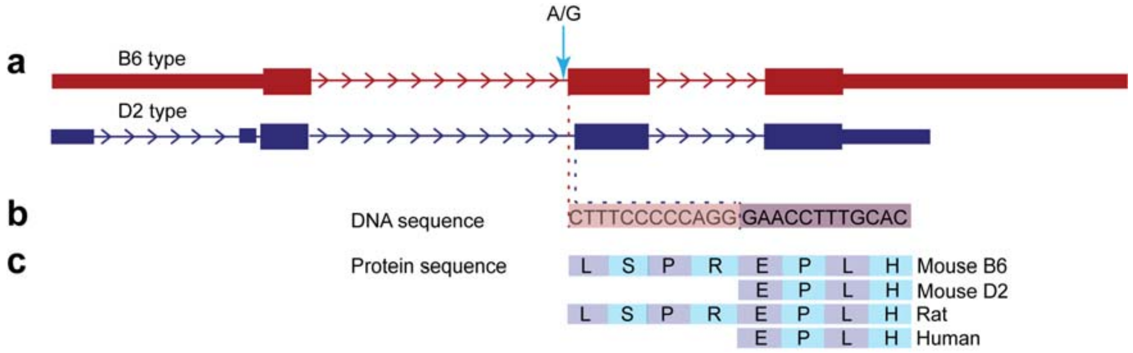

Supplementary Fig. 6. Association analysis for a frameshift variant in *Pcm1*

(a) A 1-bp deletion (T/-; chr8: at 41,309,537 bp) in *Pcm1* exon 24 was identified, supported by an mRNA isoform (accession: CF893233). The frameshift occurs in exon 24 in *Pcm1*. The deleted base is indicated by a vertical arrow. The normal amino acid sequence is shown in light purple and blue boxes. (b) *Pcm1* is statistically differentially expression in hippocampus (p = 0.03) and whole brain (p = 0.02), with higher expression from B alleles. Significance was assessed by two-tailed Students *t*-test, assuming unequal variances with two biological replicates (i.e. male and female). (c) Pearson correlation between *Pcm1* mRNA expression in hippocampus (Probe set: 1436908_at) and locomotor activity after paraoxon organophosphate (OP) acetylcholinesterase inhibitor response (GN ID 10571) in BXD family. The mRNA expression in hippocampus was measured using Affymetrix M430 2.0 microarrays. The numbers above the dots represent BXD strain name. Two parental strains, B6 and D2, are highlighted in blue and brown, respectively. (d) Human phenome scan for association of *Pcm1* (rs201598166) across BioVU. Each point represents the –log10(p value) of a single SNP-phenotype association. The horizontal red dotted line represents p = 0.01.

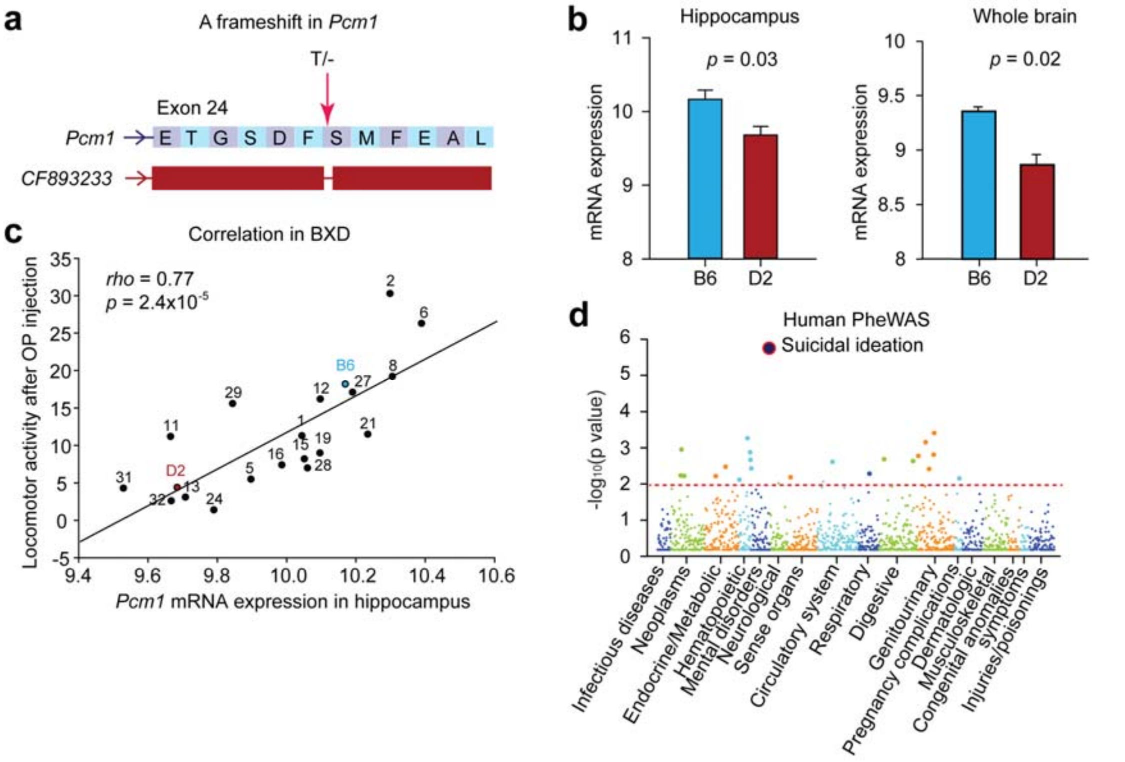

Supplementary Note 1

SNP detection and distribution

A total of 1.548 million SNPs are located in genes, including introns (95.91%), exons (2.00%), 3′ untranslated regions (UTRs) (1.83%), and 5′ UTRs (0.26%) (**Supplementary Fig. 1c**). As expected from previous work[1], SNP density varies at least 100-fold across the genome—from 50 to 5,000 SNPs/Mb.

Noncoding SNPs

All noncoding SNPs (ncSNPs) segregating within the BXD cohort were assigned a potential impact score from 0 to 1 based on locations within evolutionarily conserved elements, transcription factor binding sites, enhancers, or silencers (see **Online Methods**). Approximately 82.6% of the 4.625 million ncSNPs had negligible potential impact, whereas ~800,000 had a higher score above the mean of 0.19, and ~5000 had scores >0.9 (**Supplementary Data 9)**. As expected, ncSNPs in UTRs have comparatively high scores (5′ UTR: 0.25 ± 0.0029; 3′ UTR: 0.23 ± 0.0013) (**Supplementary Fig. 1d**).

5′ UTR variants are of particular interest because they may control mRNA transcription, stability, or translational efficiency[2]. Seventy-four ncSNPs with high-impact scores (>0.9) located in the 5′ UTR potentially alter the binding affinity of transcription factors (**Supplementary Data 9**). One of the SNPs is in a repressor element 1-silencing transcription factor (REST) binding motif; 25 SNPs are in

transcription factor binding sites from the OREGO database; and 39 are in bidirectional promoter regions for co-regulated genes.

SNP validation

We resequenced all 47 nonsense variants with PCR products and a subset of 59 randomly selected missense variants to assess the false positive rates (FPR). We validated 42 nonsense variants and 57 missense variants—an FPR of 13% and 3.38% respectively (**Fig. 2e**). Although the FPR of these variants is significantly higher than that of SNPs in general, most of these coding SNPs are true variants with potentially substantial effects on mRNA stability and protein function.

We selected 53 out of 69 splice-site variants for further validation by Sanger resequencing, and 52 were confirmed as true variants, indicating that the false positive rate was only ~2% (1/53). In addition, 26 of the ncSNPs are predicted to alter processed miRNA sequence (**Supplementary Data 10**).

Small insertions and deletions

Small insertions and deletions (indels) are more frequent than large indels (**Supplementary Fig. 2a and 2b**). Most small indels—63% of all deletions and 64% of all insertions—are 1 to 3 bp in length (**Supplementary Fig. 2a and 2b**). Single base pair indels account for 41% of small deletions and 41% of small insertions. Like SNPs, small indels are unevenly distributed due to the complex history of the parental strains and the retention of long intervals that are almost

identical by descent. Of these, 31 gave reliable PCR products and 26 frameshift mutations were validated, indicating a false positive rate of ~16% (5/31).

Large insertions and deletions

We detected 9,347 large indels using the BreakDancer program and Illumina sequence data, and 9,201 large indels using the ABI SOLiD indel pipeline. Only 1,211 indels were detected by both (16% of deletions and 13% of insertions; **Supplementary Fig. 2c and 2d**)—a remarkable discordance that is probably caused by using libraries with different insert sizes for these platforms. Large indels ranged from 100 to 98,000 bp for SOLiD reads and 100 to 970,000 bp for Illumina reads. The distribution peaks at 200–250 bp and 6700–7000 bp—the ranges expected for B1/2 SINE and L1 LINE elements, respectively (**Supplementary Fig. 2a and 2b**). A total of 162 large indels spanned exons.

The false positive rate for large deletions is high[3]. To overcome this, we used two different detection approaches: unmapped/split-read (Pindel) and anomalously mapped read pairs (BreakDancer and the ABI large indel pipeline). We detected 716 large indels that were identified using both of these approaches. These high-confidence indels range in size from 123 bp to 42,144 bp, and 26 of them cause the complete deletion of 11 genes and the additional deletion of at least one coding exon in 7 other genes. Most of these genes have unknown functions.

We also performed PCR-based validation of 20 predicted large deletions and 20 predicted insertions. A total of 32 indels were validated, a ~20% false positive rate for detecting large indels.

Effect of noncoding SNPs on the structure of miRNA

In order to identify potential SNPs in miRNA encoding genes in mouse genome, we downloaded 608 mouse pre-miRNA from the miRbase database (Release 15; http://www.mirbase.org/), and found 26 SNPs in 21 different pre-miRNAs (**Supplementary Data 10**). The pre-miRNA contains different domains with different functional significance. To gain insight into the potential functional importance of the identified polymorphisms, we mapped the SNPs to five different domains of the pre-miRNAs: (*a*) the seed region, (*b*) the mature region excluding the seed region (MIRΔseed), (*c*) the stem region complementary to the MIR (MIR*), (*d*) the stem region that is neither the MIR nor MIR*, and (*e*) the loop region. Only two miRNAs (mmu-mir-1931 and mmu-mir-1934) have SNPs within the seed region. Overall, over 95% of mouse pre-miRNAs are not affected by SNPs, and most (24/26) of observed SNPs are not within the seed region.

Comparative genomic analysis for predicted deleterious SNPs

Each SNP was evaluated in the context of conservation of the corresponding position in orthologous sequences from 77 complete genomes of higher eukaryotes. SNPs in invariable position in all mammals and/or beyond this taxonomic class were considered as likely deleterious, whereas SNPs in variable

positions in mammals and especially in rodents and primates (that together with rodents belong to the superoder *Euarchontoglires*) were considered unlikely deleterious, especially when the same or similar amino acid substitution was present in a close relative. For example, an apparently mild substitution of alanine to serine in position 404 in the *Klhl41* protein is considered likely deleterious, because Ala404 is invariable not only in mammalian orthologs, but also in orthologs from birds, amphibian, and fish. On the other hand, an apparently drastic change from cysteine to tyrosine in position 155 in the Srfbp1 protein is considered highly unlikely to be deleterious, because this position is variable in rodents and primates and the same (Cys to Tyr) or similar (Cys to His) substitutions are found in orthologs from other rodents.

To further investigate the functional consequence of these 258 deleterious SNPs as well as 63 nonsense SNPs, we performed comparative genomic analysis and identified four nonsense and 11 missense SNPs known to be associated with various diseases including inherited blindness, glaucoma, coronary artery disease, Brugada syndrome, pulmonary disease, hypomyelination, and myopathy (**Supplementary Data 6**). On the other hand, we also identified four nonsense and 12 missense SNPs predicted to be deleterious by both PolyPhen2 and SIFT that are not supported by comparative sequence analysis (**Supplementary Data 7**).

Missense variants confirmed by mass spectrometry-based proteomics data

To characterize missense variants supported by mass spectrometry-based proteomics data from the B6 and D2 strains, we created a customized protein

database containing all mouse proteins from SwissProt/trEMBL and proteins with 11,355 missense variants. A total of 189,101 MS/MS spectra from 10 fractions were searched against this database using SEQUEST (version 27 revision 13). A total of 49,639 unique peptides and 6,732 protein groups were identified at an FDR of 1%. Of these, 79 unique peptides harboring missense variants were identified (**Supplementary Data 8**).

Summary of expression phenome

A total of 84 expression datasets were used for calculating the number of mRNA assays in **Fig. 1b**, including 28 tissues (**Supplementary Data 2**). Among these, 16 datasets highlighted in grey were further used for molecular phenome scan analysis. These datasets are accessible from our website www.genenetwork.org.

PheWAS analysis using GeneNetwork

The PheWAS analysis can be performed in GeneNetwork (www.GeneNetwork.org) by simply following these three steps. We use an example of a missense variant (Chr2: 25,398,350) in *Entpd2*: Step 1: Find a marker closest to the high impact sequence variant of interest identified by our deep resequencing of the D2 genome. We can search for the closest marker located on Chromosome 2 at 25.39 Mb by searching the BXD Genotype data set using a string such as “Position=(Chr2 25 26)”, where 25 and 26 are the proximal and distal search regions on Chr 2 measured in megabases. This will return a list of markers—mainly SNPs and microsatellites—close to the high impact

sequence variants in *Entpd2*. From the list of markers, we selected rs8250941, located within ~100 Kb of the high impact sequence variants. Step 2: Compute correlations between this marker and all ~4000 BXD phenotypes. In the case of rs8250941, GeneNetwork traits 10015 and 10014 are highly correlated with this marker and have –logP association scores larger than 10. Traits that have peak association scores (LRS or LOD) that are very close to the location of sequence variants are candidate traits. Step 3: Compute correlations between the marker and all or a subset of relevant transcriptome or proteome data sets. This may produce large output tables with as many as 20,000 endophenotypes. However, these can be sorted easily by the position of the peak association score (click on column that is labeled *Max LRS Location*). When correctly sorted, this table will highlight those candidate mRNAs that presumably map to sequence variants at the *Entpd2* locus.

Examples of PheWAS analysis

The example illustrated above involves missense variants—R149Q and A297T—in ecto-nucleoside triphosphate diphosphohydrolase 2 (*Entpd2*; **Supplementary Fig. 4a**). These variants in the triphosphatase domain are linked to differences in mRNA (fold-difference = 0.6, p value <.03) and protein levels (fold-difference = 1.7, p <.01) (**Supplementary Fig. 4b**) and generate strong cis eQTLs in multiple tissues (e.g., lung, $q = 4.9\times10^{-9}$). A phenome scan highlights two enzymatic phenotypes: (1) $Ca2^{+}$- and (2) $Mg2^{+}$-stimulated ecto-ATPase activity (GN ID 10014 and 10015; $q = 9.97\times10^{-5}$ and .007) (**Supplementary Fig. 4c**). Both are

direct measures of ATPase activity—*prima facie* evidence that one or both of these SNPs are causal. In the BioVU human clinical cohort, rs34618694 in *ENTPD2,* is associated with microophthalmia (p = 2.4x10^{-4}) and visual defects (p = 2.2x10^{-3}; **Supplementary Fig. 4d**).

Among the 69 splice-site ncSNPs, an acceptor site mutation within intron 1 of *Hcfc1r1* (host cell factor C1 regulator) was of particular interest (**Supplementary Fig. 5a**). This mutation (A/G; Chr 17 at 23,674,533 bp) disrupts a cryptic AG splice acceptor site of the *B*-type isoform (**Supplementary Fig. 5a**) and creates an alternative *D* isoform that trims away four amino acids (SLSP) on the N-terminus of exon 2 (**Supplementary Fig. 5b**). The shorter *D* isoform is relatively highly conserved among mammals, including humans (**Supplementary Fig. 5c**). Among the BXD cohort, this splice acceptor variant is linked with expression of *Hcfc1r1* in the kidney (q = 5.2×10^{-3}) and differential usage of exon 2 (6-fold, high *B*) in striatum (q = 3.0×10^{-66}). The variant is also associated with the proportion of Bacillales—an order of gram-positive bacteria that include *Bacillus*, *Listeria*, and *Staphylococcus*—in the gut microbiome (GN ID 16283; q = 0.023).

Among the 27 validated frameshift variants identified, a gene involved in cell cycle regulation—pericentriolar material 1 (*Pcm1*)—is of interest due to the presence of a 1-bp deletion (Chr8:41,309,537) in *D* relative to *B* in exon 24 (**Supplementary Fig. 6a**). The mutated isoform is supported by an mRNA isoform (accession: CF893233). *Pcm1* is highly expressed in several CNS tissues, including hippocampus and striatum, and has higher expression from *B*

alleles (**Supplementary Fig. 6b**). SNP *rs3696853* within *Pcm1* is strongly linked to expression of *Pcm1* in hippocampus (q = 1.3×10^{-11}; **Supplementary Fig. 6c**). *Pcm1* is thought to be downstream of mutations in the schizophrenia susceptibility candidate gene *Disc1*[4] and may be involved in regulation of protein kinase C activity[5] and to protein kinase calcium/calmodulin-dependent kinase II beta localization to the centrosome during dendrite patterning[6]. Several studies suggest a role for *Pcm1* variation in CNS development[7], schizophrenia[8], and Huntington's disease[9,10]. As such, *Pcm1* may be relatively novel but is also a key intracellular signaling modulator that critically influences behavior and brain function. Matched analysis of the BioVU cohort shows that rs7009117 in *PCM1* is associated with psychiatric disorders, including schizophrenia (p = 6.3×10^{-4}), psychosis (p = 8.6×10^{-4}), and autism (p = 2.5×10^{-2}) (**Supplementary Fig. 6d**).

SUPPLEMENTARY REFERENCES:

1. Waterston, R.H. *et al.* Initial sequencing and comparative analysis of the mouse genome. *Nature* **420**, 520-62 (2002).
2. Hughes, T.A. Regulation of gene expression by alternative untranslated regions. *Trends Genet* **22**, 119-22 (2006).
3. Hormozdiari, F., Alkan, C., Eichler, E.E. & Sahinalp, S.C. Combinatorial algorithms for structural variation detection in high-throughput sequenced genomes. *Genome Research* **19**, 1270-1278 (2009).
4. Eastwood, S.L., Walker, M., Hyde, T.M., Kleinman, J.E. & Harrison, P.J. The DISC1 Ser704Cys substitution affects centrosomal localization of its binding partner PCM1 in glia in human brain. *Hum Mol Genet* **19**, 2487-96 (2010).
5. Chakravarthy, B., Menard, M., Brown, L., Atkinson, T. & Whitfield, J. Identification of protein kinase C inhibitory activity associated with a polypeptide isolated from a phage display system with homology to PCM-1, the pericentriolar material-1 protein. *Biochem Biophys Res Commun* **424**, 147-51 (2012).
6. Puram, S.V. *et al.* A CaMKIIbeta signaling pathway at the centrosome regulates dendrite patterning in the brain. *Nat Neurosci* **14**, 973-83 (2011).

7. Ge, X., Frank, C.L., Calderon de Anda, F. & Tsai, L.H. Hook3 interacts with PCM1 to regulate pericentriolar material assembly and the timing of neurogenesis. *Neuron* **65**, 191-203 (2010).
8. Gurling, H.M. *et al.* Genetic association and brain morphology studies and the chromosome 8p22 pericentriolar material 1 (PCM1) gene in susceptibility to schizophrenia. *Arch Gen Psychiatry* **63**, 844-54 (2006).
9. Engelender, S. *et al.* Huntingtin-associated protein 1 (HAP1) interacts with the p150Glued subunit of dynactin. *Hum Mol Genet* **6**, 2205-12 (1997).
10. Keryer, G. *et al.* Ciliogenesis is regulated by a huntingtin-HAP1-PCM1 pathway and is altered in Huntington disease. *J Clin Invest* **121**, 4372-82 (2011).
